# Perceived social support and pre-service teachers' career calling: roles of vocational outcome expectations and professional identity

**DOI:** 10.3389/fpsyg.2026.1826997

**Published:** 2026-06-26

**Authors:** Weiwei Shang, Yilin He, Bing Bai

**Affiliations:** 1 School of Marxism, East China Normal University, Shanghai, China; 2 School of Marxism, Chongqing University, Chongqing, China

**Keywords:** career calling, perceived social support, pre-service teachers, professional identity, vocational outcome expectations

## Abstract

**Introduction:**

Achieving sustainable development goal 4 in education hinges on teachers' sustainable career development, in which career calling serves as an endogenous driving force. This study investigated how perceived social support, vocational outcome expectations, professional identity and career calling were related among pre-service teachers.

**Methods:**

A questionnaire survey was conducted among 1,029 Chinese pre-service teachers. Structural equation modeling (SEM) was employed to analyze the relationships among perceived social support, vocational outcome expectations, professional identity, and career calling.

**Results:**

The results revealed that perceived social support was significantly and positively related to vocational outcome expectations, professional identity and career calling. Furthermore, vocational outcome expectations and professional identity mediate the relationship between perceived social support and career calling both individually and in series.

**Discussion:**

Our findings highlight that social support constitutes a key factor in promoting career calling among pre-service teachers. Implications include the need to construct a multi-dimensional social support system to strengthen pre-service teachers' vocational outcome expectations and professional identity, thereby facilitating the development of their career calling.

## 1 Introduction

Teachers are the key to achieving equitable quality education, as outlined in Sustainable Development Goal 4 (SDG 4). Discussions and actions surrounding SDG 4.c increasingly focus on the “quality” of the teaching workforce and the “sustainable development” of the teaching profession. Sustainable career development is conceptualized as the attainment of unity among adaptability, resilience, and meaning within one's occupational psychology domain. Career calling (CC), in this regard, acts as the fundamental source that concurrently nourishes all three dimensions. As such, it constitutes the endogenous driving force behind sustainable career development and represents an essential psychological resource for maintaining sustained occupational engagement over the long term. The study of CC has gradually become a hot topic in the field of occupational psychology and positive psychology research. As an enabler of career development, individuals with high CC tend to have higher work and life satisfaction, happiness, career adaptability, career maturity, career commitment, and work engagement (Hagmaier and Abele, 2012; Duffy and Dik, 2013; Vianello et al., 2018; Duffy et al., 2011; Parola et al., 2023; Marsh and Dik, 2026). At the same time, CC also brings social benefits due to its pro-social nature (Ni et al., 2024). Importantly, career calling is not an innate trait; longitudinal evidence and investigations into its antecedents have consistently demonstrated that CC is a dynamic and malleable construct (Bott and Duffy, 2015; Dobrow, 2013; Dobrow and Heller, 2015). Accordingly, fostering and strengthening career calling is critical for promoting individuals' long-term career sustainability. Within the spectrum of professional groups, teachers undertake the sacred mission of knowledge transmission and generational education. For pre-service teachers preparing to enter the profession, developing a clear sense of career calling is particularly vital for enhancing their career resilience and professional self-regulation (Dumulescu et al., 2015). However, current empirical research exploring the antecedents of CC remains relatively limited (Zhang and Guo, 2023). According to Social Cognitive Career Theory (SCCT; Lent et al., 1994), personal and social factors shape individual's outcome expectations and goal intentions, which ultimately predict behavioral performance (Lent et al., 2008; Hall and Chandler, 2005). Recent empirical evidence has demonstrated that perceived social support can positively predict career calling via professional identity (Zhang and Guo, 2023). However, the mechanism by which perceived social support influences career calling through the serial roles of vocational outcome expectations and professional identity remains underexplored. To further clarify the impact of perceived social support on career calling and unpack the developmental mechanism of career calling among Chinese pre-service teachers, the present study examines the key roles of vocational outcome expectations and professional identity within the SCCT framework. This study contributes to a more comprehensive understanding of the critical factors underlying career calling among Chinese pre-service teachers and provides empirical evidence that can inform research and practice in other cultural contexts worldwide.

## 2 Literature review and development of hypotheses

### 2.1 Career calling

The term “calling” originally originated in the religious realm (Steger et al., 2010), and refers to God's call for individuals to have meaningful, life-changing experiences that benefit others (Ponton et al., 2014). Scholars have introduced calling into the vocational realm to describe infusing an individual's work with a sense of purpose and meaning (Dik and Shimizu, 2019; Steger and Dik, 2009; Sturges et al., 2019). Work is a blessing for those who have a career as their calling (Ponton et al., 2014). As research continues, different scholars have explained the concept of vocational calling differently (Dik and Duffy, 2009; Elangovan et al., 2010; Hagmaier and Abele, 2012; Hunter et al., 2010; Bunderson and Thompson, 2009; Wrzesniewski et al., 1997; Dobrow and Tosti-Kharas, 2011), and a division between the classical view (associated with religion), the modern view (more secularized and self-directed), and the neoclassical view (more focused on the nature of calling) has gradually developed (Ponton et al., 2014; Zhang et al., 2015). Despite the nuances between the different definitions, it generally conveys the idea that people who make work their calling are not only concerned with financial rewards or career advancement, but also more focused on the enjoyment of fulfilling and socially useful work (Wrzesniewski et al., 1997). Of these, Dik and Duffy (2009) definition is the most widely influential, defining CC as “a transcendent summons, experienced as originating beyond the self, to approach a particular life role in a manner oriented toward demonstrating or deriving a sense of purpose or meaningfulness and that holds other-oriented values and goals as primary sources of motivation.” In this definition, career calling has three dimensions, the first of which is transcendent summons from external sources such as God and social needs; the second relates to the purpose and meaning of people's work, and the third is pro-social behaviors that contribute to the wellbeing of the society in some positive way. These triple dimensions are also reflected in the Chinese college student population and have a new connotation of following parents' wishes and arrangements and emphasizing social contribution in the Chinese social context under the traditional Chinese values of collectivism and filial piety (Zhang et al., 2015). Currently, research on the antecedents and consequences of CC has attracted the interest of an increasing number of scholars. Reviewing the existing literature, scholars have explored CC predictors at both individual and social factor levels. Undeniably, a wide range of intrinsic drives and personality dispositions have been identified as prerequisites for professional vocation (Zhang and Guo, 2023). Among them, personal factors such as Prosocial Work Motivation (Dik et al., 2012), Life Meaning (Zhang et al., 2017), Career Decision Self-efficacy, Personal Growth Initiative (Bott and Duffy, 2015), Self-Reflection (Xie et al., 2016; Ran et al., 2023), Self-Concept (Lau et al., 2020), Career Goal Self-efficacy (S. Zhang et al., 2022), Core Self-Evaluation (Shen et al., 2021), Character Strength (Harzer and Ruch, 2012; Lian et al., 2021)were significant predictors of CC. However, the influence of environmental factors on CC still needs to be deepened, and in particular, relatively few studies have been conducted on social factors as predictors of CC and the intrinsic mechanism of action. Therefore, our study attempts to introduce Social Cognitive Career Theory to explore how perceived social support influences CC through vocational outcome expectations and professional identity, enriching research on CC predictors.

### 2.2 Perceived social support and career calling

Social support is defined as the social resources that persons perceive to be available or that are actually provided to them by non-professionals in the context of both formal support groups and informal helping relationships (Gottlieb and Bergen, 2010). It can be divided into received social support and perceived social support (PSS). Unlike received social support which refers to the social support that an individual actually receives (Eagle et al., 2019), PSS refers to the cognitive appraisal of the fact that social relationships will provide resources such as emotional support (Barrera Jr, 1986; Cohen, 1992). It emphasizes the individual's subjective feelings or experiences of social support (Xia et al., 2012). Numerous studies have showed that high levels of PSS lead to higher wellbeing (Cohen and Wills, 1985; Diener and Fujita, 1995) and satisfaction (Gan et al., 2020; Gutiérrez et al., 2017), reduce negative emotions such as depression (Eagle et al., 2019)and loneliness (Salimi and Bozorgpour, 2012), as well as bringing about positive behavioral performance such as creativity enhancement (Tan et al., 2022). SCCT affirms the role of an individual's cognitive appraisal of the environment in the influence of specific situational factors on choice behavior and suggests that there is an important role of environmental perceptions on occupational behavior. According to SCCT, PSS has a direct and powerful impact on the formation and implementation of choices as an important situational factor, which makes it possible that people who perceive more social support may perform better in career development than those who perceive less social support. Furthermore, the literature provides evidence that PSS is positively related to an individual's career development. Ghosh and Fouad (2017) found that social support was a positive predictor of career adjustment. A study conducted by Karatepe and Olugbade (2017) among hotel employees in Nigeria found that workplace social support has a positive impact on occupational adaptation. Chan (2020) found that there was a positive predictive effect of social support on career beliefs. In addition, there is relevant empirical evidence for a positive correlation between PSS and CC, and studies have confirmed that social development (Dalla Rosa et al., 2019; Zhang and Guo, 2023), community social capital (Su et al., 2024), Family influence (Tian et al., 2021; Zhang and Zhang, 2022), supervisor support (Li et al., 2021) contribute to the development of CC. Shang et al.'s study of Chinese pre-service teachers showed that adequate material support, sound professional support, and diversified emotional support contributed to the development of pre-service teachers' career calling (Shang et al., 2022). Liu et al. (2024) showed that Parent-Initiated Support had a positive impact on teachers' career calling. Bott et al. (2017) conducted a qualitative study among physicians and found that support from family and friends significantly fostered their commitment to their CC. Therefore, we propose the first hypothesis:

Hypothesis 1: There is a positive effect of perceived social support on career calling among pre-service teachers.

### 2.3 The mediating role of vocational outcome expectations

Vocational outcome expectations is an important concept in SCCT, which is an individual's imagined outcome of performing a particular behavior (Bandura, 1977, 1986; Lent et al., 1994), and is often referred to in related research in the field of career development as Vocational Outcome Expectations (VOE), which describes an individual's beliefs about the consequences of a particular occupational behavior, they may include material outcomes (e.g., money), social outcomes (e.g., social status, recognition by parents, friends, and other significant others), and self-evaluative outcomes (e.g., self-satisfaction, intrinsic motivation), which all have a significant impact on occupational behavior (Lent et al., 1994; Lu et al., 2024). According to SCCT, PSS, as one of the important environmental factors, has a positive predictive effect on VOE. A growing body of research suggests that individuals with higher levels of social support are able to achieve better outcomes in career choice and career development (Xia et al., 2020; Wang et al., 2024; Jemini-Gashi et al., 2021). Isik (2013) showed that perceived social support from family, friends, and significant others was significantly and positively related to vocational outcome expectations. A study of Turkish students by Aslan and Koçak, 2025 showed that perceived social support was a positive and significant predictor of vocational outcome expectations. A study conducted by Wang et al. (2023) in China showed that the level of social support has a significant effect on prestige and welfare stability in career expectations. Meanwhile, according to the SCCT, vocational outcome expectations affect individuals' learning engagement and occupational performance (Brown et al., 2011), and positive outcome expectations (e.g., high rewards, high social status) may motivate individuals to take positive actions that lead to better occupational performance. Shang et al. (2022) showed that vocational outcome expectations were positively related to learning engagement in a study of pre-service teachers. expectations are positively related to learning engagement. Therefore, as a positive occupational performance, vocational outcome expectations is likely to have a positive predictive effect on CC. Those with positive vocational outcome expectations tend to be more engaged, passionate, and find the purpose and meaning in their work (Dalla Rosa et al., 2019; Zhang and Guo, 2023). However, whether these influencing factors affect pre-service teachers' career calling remains unclear. Accordingly, the second hypothesis can be proposed:

Hypothesis 2: Vocational outcome expectations mediate the relationship between perceived social support and career calling among pre-service teachers.

### 2.4 The mediating role of professional identity

In addition to vocational outcome expectations, the role of professional identity (PI) in the perceived social support-career calling relationship also deserves attention. Often regarded as a key construct for teachers‘ professional development (Zhang et al., 2016), professional identity is a consistent, continuous, and integrated perception of oneself in the work environment (Erikson, 1956), which concerns an individual's career in terms of his/her attributes, beliefs, values, motives, and experiences and tends to be highly malleable early in an individual's career (Ibarra, 1999). High levels of PI tend to be associated with a clear self-concept (Park and Moon, 2022), interests, goals (Holland et al., 1980) and values (Li et al., 2015). Numerous studies have shown that social support has a positive impact on the formation of professional identity. A study on a group of teachers in China by Zhang and Guo (2023) supported the positive impact of perceived social support on professional identity. A study of Chinese technical college students by Zhang et al. (2021) found that professional identity mediated the relationship between occupationally related parental support and occupational adaptability. Chemers et al. (2011) found that professional identity mediated the relationship between science support activities and occupational commitment in a study of American students. Zhu and Cheng (2024) found that social support directly and positively predicted rural teachers' professional identity in a study of rural teachers in China. Zhang et al. (2015) showed that pre-service teachers in China were provided with social support from significant others, such as internship supervisors, enhanced their identification with the teaching profession Meanwhile, the positive work outcomes resulting from PI have also received attention from a wide range of scholars. A range of studies have shown that individuals with a strong professional identity tend to have higher work engagement (Sun et al., 2022) and view teaching as a long-term passion rather than just a job (Wu et al., 2024), and are more likely to have a better understanding of their roles and responsibilities (Ye and Zheng, 2017). Galles and Lenz (2013) study showed that professional identity contributes to the formation of career calling. Dalla Rosa et al. (2019) study similarly showed that a clear professional identity and active engagement in learning had a positive impact on career calling. Zhang and Guo (2023) study further suggests that professional identity may mediate the relationship between perceived social support and career calling. Therefore, the hypothesis of our study is proposed:

Hypothesis 3: Professional identity mediates the relationship between perceived social support and career calling among pre-service teachers.

### 2.5 Serial mediation through vocational outcome expectations and professional identity

As mentioned above, vocational outcome expectations and professional identity are important links between perceived social support and career calling. Some studies have found an association between vocational outcome expectations and professional identity, which raises the question of whether there is a chain mediation between PI and CC. According to the SCCT, external environmental factors will likely influence an individual's goal intention and ultimately their behavioral performance through vocational outcome expectations (Lent et al., 1994). Therefore, the environment will likely influence an individual's professional identity through vocational outcome expectations. Yao et al. (2020) showed that vocational outcome expectations mediated the relationship between policy satisfaction and professional identity, and that vocational outcome expectations directly influenced teachers' professional identity. Gao and Yang (2022) showed that vocational outcome expectations mediated the relationship between organizational psychosocial support and professional identity in a study of Chinese doctoral students. in the relationship between organizational psychological support and professional identity. Accordingly, it is reasonable to infer that individuals with higher levels of perceived social support are able to have more positive vocational outcome expectations, which leads to higher levels of professional identity, and thus are more likely to regard their work as calling. Therefore, our research formulates the hypothesis:

Hypothesis 4: Perceived social support can influence career calling through the chain-mediated effects of vocational outcome expectations and professional identity among pre-service teachers.

**Figure 1:**
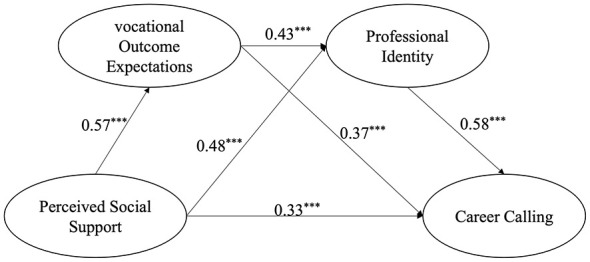
Hypothesized model of mediated relationships between perceived social support on career calling.

Hypotheses 1–4 are exploratory and reflect the fact that research on pre-service teachers' learning engagement in China is still at an early stage. We used the SCCT framework to further understand the impact of perceived social support on career calling of pre-service teachers. More specifically, this study aimed to examine the internal mechanism by which perceived social support affects career calling through the mediating roles of vocational outcome expectations and professional identity. Furthermore, it aimed to provide a solid theoretical rationale for improving pre-service teachers' career calling and the sustainable development of their profession. Figure 1 depicts the research model.

## 3 Materials and methods

### 3.1 Participants and procedure

Data were gathered using an online survey hosted on the Wenjuanxing platform. The link shared with participants contained the questionnaire and instructions to complete it. Also included were guidelines informing teachers that their participation would be voluntary, anonymous, and confidential, with no time limit for completing the questionnaire. These procedures increased the credibility of the self-report data for representing the facts of the situation and the genuine sentiments of the respondents. The participants were recruited from East China Normal University (Shanghai), a leading center of teacher education in China that features in the central government's Double First Class University Plan.

A total of 1,078 students voluntarily completed, with 1,029 valid responses received after removing duplicate or contradictory replies, a rate of 95.45%. Participants ranged in age from 17 to 23 years (*M* = 19.62; *SD* = 4.59; 73% girls). There were 237 freshmen (23%), 325 sophomores (31.6%), 316 juniors (30.7%), and 151 seniors (14.7%). Among these, 652 students (63.4%) were from rural backgrounds, and 377 (36.6%) were from cities.

### 3.2 Measures

#### 3.2.1 Career calling

Career calling was assessed using the 12-item Calling Scale (Dobrow and Tosti-Kharas, 2011). The questionnaire was translated into Chinese by a graduate student majoring in English. Respondents were asked to rate their agreement with such statements as “I am passionate about being a teacher”, “I enjoy being a teacher more than anything else,” and “Being a teacher is a deeply moving and gratifying experience for me.” A Likert 5-point scale was used (from 1 for “strongly disagree” to 5 for “strongly agree”), and, with higher scores representing the higher career calling of teachers. The scale also has good reliability in the Chinese cultural context (Zhang et al., 2015). In this study, the internal consistency of our research was found to be strong (α = 0.91).

#### 3.2.2 Perceived social support

The perceived social support scale developed by Zimet et al. (1988) and revised by Wang et al. (1999) was used to reflect the social support received by pre-service teachers. The questionnaire consists of 12 items, divided into 3 subscales: (1) family, containing 4 items, such as “My family tries to help me” (α = 0.81); (2) friends, containing 4 items, such as “I have friends with whom I can share my joys and sorrows” (α = 0.82);(3) significant other, containing 4 items, such as “There is a special person who is around when I am in need” (α = 0.85). The participants rated the items on a 7-point Likert-type response format (1 = very strongly disagree; 7 = very strongly agree). The higher the score, the better the social support received by the individual. The questionnaire has good reliability and validity in the Chinese context (Kong et al., 2012). In this study, the internal consistency of the scale, measured by the Cronbach alpha coefficient, was 0.84.

#### 3.2.3 Vocational outcome expectations

The Vocational Outcome Expectations Scale - Revised (VOER) developed by McWhirter et al. (2000) and revised by Metheny and McWhirter (2013) was used to assess vocational outcome expectations of pre-service teachers. The Chinese translation of the 12-item scale was administered in this study. Respondents were asked to rate their agreement with such statements as “The outcomes of my career plan are satisfactory to me.” A Likert 5-point scale was used (from 1 for “strongly disagree” to 5 for “strongly agree”), and, with higher scores representing the more positive outcome expectations of pre-service teachers. The scale also has good reliability in the Chinese cultural context (Yao et al., 2020). In this study, the internal consistency of our research was found to be strong (α = 0.92).

#### 3.2.4 Professional identity

Pre-service teachers' professional identity was assessed using the 15-item Teacher Professional Identity Scale developed in the Chinese cultural context (TPI; Zhao et al., 2011). Respondents were asked to rate their agreement with such statements as “After graduation, I will choose to become a teacher”. A Likert 5-point scale was used (from 1 for “strongly disagree” to 5 for “strongly agree”), and, with higher scores representing the higher professional identity of pre-service teachers. The questionnaire has good reliability and validity in the Chinese context (Yao et al., 2020). Cronbach's alpha for TPI in the current study was 0.89.

### 3.3 Data analysis

The analytic strategy proceeded in three stages. All statistical analyses were performed using SPSS 22.0 and AMOS 22.0 software. First, following the procedure described by Podsakoff et al. (2003), we employed Harman's single-factor test to check for common method bias. Subsequently, descriptive statistics and correlation analyses were performed using SPSS, with means and standard deviations reported. Next, the measurement model was first analyzed to examine whether each of the four latent constructs was well represented by its indicators. To increase the reliability of the parameter estimates, three item parcels were created as indicators of materialism. Using AMOS 22.0, maximum likelihood estimation was used to analyze the structural model. In the structural equation modeling, the direct effect of perceived social support on career calling was first tested. Second, a mediated model was tested; this contained a direct path from perceived social support to career calling and one mediated by vocational outcome expectations and professional identity. In line with Hu and Bentler (1999), the model fit was generally considered acceptable when the RMSEA and SRMR values were below 0.08 and the GFI, IFI, and CFI values were above 0.90. Finally, the statistical significance of the pathways and indirect effects in each model were examined using bootstrapping to calculate the bias-corrected percentile confidence intervals.

## 4 Results

### 4.1 Common-method bias test

To control for common method bias, Harman's single-factor test were used (Eby and Dobbins, 1997). Among the factors, eight had eigenvalues greater than one, with the first factor explaining 27.13% of the total variance, well below the recommended threshold of 40%. Furthermore, the confirmatory factor analysis (CFA) conducted via the method-factor approach showed that the model did not fit the data closely (*X*^2^/df = 18.323, CFI = 0.483, IFI = 0.933, RMSEA = 0.053). These investigations confirmed the absence of serious common method bias in the data.

### 4.2 Correlation analyses

The Spearman's correlations for the means and SDs of all study measures are presented in Table 1. As the table shows, perceived social support was positively correlated with career calling (*r* = 0.382, *p* <0.01), vocational outcome expectations (*r* = 0.523 *p* <0.01), and professional identity (*r* = 0.473, p <0.01). Moreover, both vocational outcome expectations and professional identity were positively related to career calling, with values ranging from moderate to large (0.478 to 0.637). Similarly, vocational outcome expectations were positively correlated with professional identity (*r* = 0.328, *p* <0.01).

**Table 1:** Means, standard deviations and correlations.

Measures	*M*	*SD*	1	2	3	4
1.PSS	3.01	0.482	1.000			
2.VOE	3.78	0.573	0.523^**^	1.000		
3.PI	3.66	0.532	0.473^**^	0.328^**^	1.000	
4.CC	3.31	0.528	0.382^**^	0.478^**^	0.637^**^	1.000

^**^p <0.01; *N* = 1,029, *pre-service teachers; CC, career calling; PSS, perceived social support; VOE, vocational outcome expectations; PI, professional identity*.

### 4.3 Structural equation modeling

**Figure 2:**
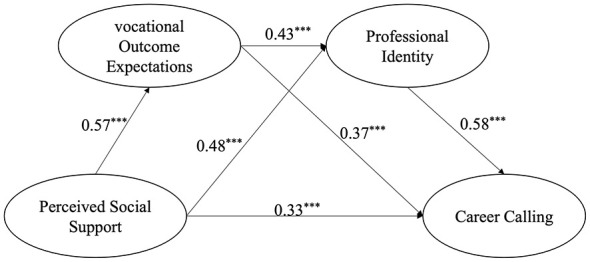
The serial mediation model with vocational outcome expectations and professional identity as mediators of the linkage between perceived social support and career calling. ***P <0.001.

AMOS 22.0 software was employed to conduct structural equation modeling (SEM) for the hypothetical model illustrated in Figure 1. First, the direct effects of the variables of perceived social support, vocational outcome expectations, professional identity, and career calling were tested. A series of path analyses were performed with gender and educational degree included as control variables, and the model fit indices (X^2^/df = 2.33, CFI = 0.97, TLI = 0.93, RMSEA = 0.067, SRMR = 0.058) indicated a good fit between the hypothetical model and the observed data. As presented in Figure 2, all direct path coefficients were statistically significant in the hypothesized directions. Specifically, perceived social support exerted a significant positive effect on pre-service teachers' vocational outcome expectations (β = 0.57, *p* <0.001), professional identity (β = 0.48, *p* <0.001), and career calling (β = 0.33, *p* <0.001). Similarly, both vocational outcome expectations (β = 0.37, *p* <0.001) and professional identity (β = 0.58, *p* <0.001) demonstrated significant positive impacts on pre-service teachers' career calling. Moreover, vocational outcome expectations had a significant positive effect on professional identity (β = 0.43, *p* <0.001). Collectively, these results provide support for our first hypothesis.

To examine the indirect effects of perceived social support and career calling, we used bootstrapping with 5,000 rounds of sampling. Table 2 showed the results after controlling for age (p = 0.73) and gender (p = 0.38). The indirect effects of perceived social support on career calling through vocational outcome expectations were positive and statistically significant. Vocational outcome expectations had a mediating effect of 0.211 [95% CI (0.258, 0.357), excluding 0], accounting for 22.0% of the total effect, thereby confirming Hypothesis 2. The indirect effects of perceived social support on career calling through professional identity were positive and statistically significant. Professional identity had a mediating effect of 0.278, [95% CI (0.237, 0.578), excluding 0], accounting for 28.9% of the total effect, thereby confirming Hypothesis 3. It followed that the mediating effect of professional identity was greater. The chain mediation effect of vocational outcome expectations and professional identity in the relationship between perceived social support and career calling were positive and statistically significant, with indirect effect = 0.142, and the 95% confidence interval excludes zero (0.121, 0.383). And, the ratio of the mediating effect size to the total effect size accounted 14.8% of the total effect, thereby supporting Hypothesis 4. Overall, a chain mediation effect was verified in our proposed model and showed the significant direct and indirect effects of perceived social support on career calling through the mediation of vocational outcome expectations and professional identity.

**Table 2:** Bootstrap analyses of the significance of mediation.

Model pathways	Effect size	95% confidence interval		Percentage
		Boot LLCI	Boot ULCI	
PSS → CC	0.33	0.133	0.352	
PSS → VOE	0.57	0.187	0.398	
PSS → PI	0.48	0.162	0.377	
VOE → PI	0.43	0.158	0.373	
VOE → CC	0.37	0.147	0.303	
PI → CC	0.58	0.193	0.399	
PSS → VOE → CC	0.211^***^	0.258	0.357	22.0%
PSS → PI → CC	0.278^***^	0.237	0.578	28.9%
PSS → VOE → PI → CC	0.142^***^	0.121	0.383	14.8%

^***^p <0.001; *N* = *1,029, pre-service teachers; CC, career calling; PSS, perceived social support; VOE, vocational outcome expectations; PI, professional identity*.

## 5 Discussions

The exploration of CC antecedents is key to answering the question of how to cultivate pre-service teachers' career calling. Based on the Social Cognitive Career Theory (SCCT), our study constructed a chain mediation model aimed at exploring the relationship between perceived social support and career calling among pre-service teachers, and further exploring the mediating roles of vocational outcome expectations and professional identity in this relationship. The results of the study indicated that perceived social support directly and positively predicted career calling. Furthermore, vocational outcome expectations and professional identity were found to sequentially mediate this relationship. These findings carry several theoretical and practical implications.

### 5.1 Theoretical implications

First, the study found that perceived social support has a direct positive effect on career calling, suggesting that social support plays an important role in an individual's career development, which is consistent with the findings of previous studies (Duffy and Autin, 2013; Ghosh and Fouad, 2017; Willard and Lavallee, 2016; Dalla Rosa et al., 2019). This suggests that individuals who perceive support from family, friends, and other significant others are able to face difficulties and challenges in their professional growth with a more positive mindset, develop healthy psychological and positive work attitudes (Steger et al., 2010), and develop a higher level of occupational commitment and a more active engagement in learning, thus fostering career calling with pro-social behavioral tendencies. This finding enriches the exploration of environmental factors as predictors of career calling. It is worth mentioning that we shifted the study of calling from the work environment to the educational context, focusing on the group of pre-service teachers who are about to enter the workforce, thus demonstrating that perceived social support is equally important in the formation of pre-service teachers' career calling. For pre-service teachers, their current professional learning and development are of critical importance to their future job growth, and perceived social support serves as an individual coping resource when they encounter certain difficulties in their professional learning and pursuit of their career aspirations (Cohen and Wills, 1985), they are able to obtain comfort and encouragement from others when they are in a negative mood, which plays an important role in facilitating their professional growth and the formation and development of their career calling.

Second, we provided more evidence that vocational outcome expectations play an important mediating role in the relationship between pre-service teachers' perceived social support and career calling, supporting past findings that social support has a positive effect on vocational outcome expectations (Gushue and Whitson, 2006; Li et al., 2024; Sun et al., 2024). Meanwhile, the present study supports the SCCT's findings that environmental contextual factors can lead to positive vocational outcome expectations and play a central role in future career choices (Franco et al., 2019), which ultimately produce positive career performance. An individual with higher levels of social support, such as family, friends, and other significant others, is able to cultivate his or her career calling by having more positive vocational outcome expectations in his or her pursuit of career advancement, engaging in the workplace with a positive attitude, and finding the purpose of the work and what it means. At the same time, we also demonstrated that professional identity can play an important mediating role between perceived social support and career calling, supporting the findings of previous studies (Wu et al., 2024; Yin et al., 2024). Specifically, the support that pre-service teachers receive from family, friends, and other significant others can help them cope with stress and anxiety and reduce emotional internalization (Gao and Yang, 2022), which enables them to focus more on professional competence enhancement and to form a high level of professional identity (Praskova et al., 2015), which prompts them to find purpose and meaning in their professional development, and thus to form the career calling.

A third significant finding from this study was that perceived social support positively influences Chinese pre-service teachers‘ career calling through the chain-mediated effects of vocational outcome expectancy and professional identity. This finding both provides research support for SCCT's theoretical framework of person-environment interactions and further validates previous research findings (Gao and Yang, 2022). Research suggests that if pre-service teachers are encouraged to pursue teaching as a career (Franco et al., 2019), receive more social support, they are more likely to develop a stronger career -environmental fit, hold more positive attitudes toward learning (Kenny et al., 2003; Wall et al., 1999), and cope with employment pressures and overcome career barriers with adequate psychological preparation, thus maintaining positive vocational outcome expectations (Ma and Shea, 2021). With positive vocational outcome expectations, pre-service teachers will be more likely to gain positive perceptions of the value of the teaching profession (Guccione and Bryan, 2023), and have a clearer sense of their own occupational identification, leading to the formation of a professional identity (Hazari et al., 2010). Ultimately, pre-service teachers with a high professional identity will be more able to deeply understand the value and meaning of educational work, and develop career calling by making the teaching profession their life goal and investing a high degree of passion in it (Galles and Lenz, 2013; Praskova et al., 2015). The results of this research further enriched the theoretical understanding of SCCT and deepened the study of factors influencing career calling. Based on the findings, our study suggests that in the process of pre-service teachers' career development, emphasis should be placed on the construction of supportive environments to guide individuals to develop positive vocational outcome expectations and professional identities, thus helping them to cultivate their career calling.

### 5.2 Practical implications

The findings of this study provide important practical implications for ensuring the formation of career calling among pre-service teachers and the sustainable development of their careers. First, for higher education institutions offering teacher education programs, it is crucial to establish a multi-dimensional perceived social support system for pre-service teachers. Educational administrators and teachers should attach great importance to the positive role of social support in shaping students' career calling, and take targeted measures to strengthen the support from schools, families, and peers. Schools can set up specialized counseling centers and professional guidance teams to provide personalized career advice, emotional comfort, and resource support for normal college students, helping them perceive the care and attention from the school. Meanwhile, schools should strengthen communication and cooperation with families, guide parents to establish a positive attitude towards their children's career choice of being a teacher, provide emotional encouragement and practical support, and form a joint force between school and family support to lay a solid foundation for the formation and development of students' career calling. Second, it is necessary to focus on improving normal college students' career outcome expectation and career identity, so as to give full play to their dual mediating role in the influence of perceived social support on career calling. On the one hand, higher education institutions should optimize the curriculum system of teacher education, integrate career planning and professional development courses into the whole process of talent training, help pre-service teachers understand the development prospects of the teaching profession, clarify the value and significance of being a teacher, and enhance their confidence in the professional future and career outcome expectations. On the other hand, schools should carry out various forms of practical teaching activities, such as educational internships, teaching observation, and volunteer teaching, to provide students with opportunities to experience the teaching profession in practice, help them deeply understand the connotation of the teaching profession, enhance their sense of identity and belonging to the profession, and further promote the transformation of perceived social support into stable career calling through the improvement of career outcome expectations and career identity. Finally, for educators and related practitioners in the field of teacher education, it is necessary to pay attention to the individual differences of pre-service teachers in perceived social support, career outcome expectation, career identity, and career calling. When formulating and implementing relevant support and training measures, individualized strategies should be adopted to provide targeted guidance and support for students with different needs, so as to effectively improve the overall level of pre-service teachers' career calling, cultivate high-quality professional teachers.

### 5.3 Limitations and future research

Nevertheless, several limitations need to be acknowledged in this study. First, the sample was restricted to university pre-service teachers from a single university, lacking representation from in-service teachers in primary and secondary schools, as well as educators from other disciplines or demographic backgrounds. Future research should recruit more diverse samples across different universities, teaching stages, subject areas, genders, and regions to enhance generalizability. Second, this study adopted a cross-sectional design, which precludes causal inferences regarding the relationships among perceived social support, vocational outcome expectations, professional identity and career calling. Cross-sectional data capture only a single time point, thus failing to reflect the dynamic, reciprocal, or developmental nature of these variables. For instance, career calling may not only be an outcome but also a predictor of professional identity or perceived social support over time, but the present design cannot test such bidirectional or cyclical effects. Longitudinal, experimental, or prospective designs are warranted to verify the causal directions and temporal dynamics of these variables. For example, tracking developmental changes in career calling from teacher preparation to early career employment would help disentangle the ordering and mutual influences among the constructs.Third, the present research only examined the dual mediating mechanisms, without considering other potential factors (e.g., career interests, work meaning) that may also shape preservice teachers' career calling. Subsequent studies should incorporate these variables as mediators or moderators to construct a more comprehensive explanatory model. Fourth, all participants were recruited from mainland China, which limits the cross-cultural generalizability of the findings. Further cross-national and cross-cultural investigations are needed to test the universality of the proposed model. Finally, this study provided only a snapshot of the targeted constructs; future longitudinal and field-based investigations are encouraged to track developmental changes in career calling from teacher preparation to early career employment.

## 6 Conclusion

This study investigated the direct and indirect predictors of career calling among preservice teachers in China. The findings provide a foundational understanding of how perceived social support influences career calling through the mediating roles of vocational outcome expectations and professional identity. Drawing on Social Cognitive Career Theory (SCCT), this research extends the existing literature by elucidating the mechanisms through which perceived social support relates to career calling. Furthermore, the significant mediating effects of vocational outcome expectations and professional identity underscore the importance of enhancing the attractiveness and recognition of the teaching profession.

## Data Availability

The original contributions presented in the study are included in the article/supplementary material, further inquiries can be directed to the corresponding author/s.
